# Evaluating and Mapping of Spatial Air Ion Quality Patterns in a Residential Garden Using a Geostatistic Method

**DOI:** 10.3390/ijerph8062304

**Published:** 2011-06-20

**Authors:** Chen-Fa Wu, Chun-Hsien Lai, Hone-Jay Chu, Wen-Huang Lin

**Affiliations:** 1 Department of Horticulture, National Chung Hsing University, 250, Kuo Kuang Road, Taichung City 402, Taiwan; E-Mail: hyunhikt@hotmail.com; 2 Department of Soil and Water Conservation, National Chung Hsing University, 250, Kuo Kuang Road, Taichung City 402, Taiwan; E-Mail: wulai.studio@msa.hinet.net; 3 Department of Geomatics, National Cheng-Kung University, 1, University Road, Tainan City 701, Taiwan; E-Mail: honejaychu@gmail.com

**Keywords:** negative air ions, positive air ions, geostatistic method, Kriging, garden, air ion index

## Abstract

Negative air ions (NAI) produce biochemical reactions that increase the levels of the mood chemical serotonin in the environment. Moreover, they benefit both the psychological well being and the human body’s physiological condition. The aim of this research was to estimate and measure the spatial distributions of negative and positive air ions in a residential garden in central Taiwan. Negative and positive air ions were measured at thirty monitoring locations in the study garden from July 2009 to June 2010. Moreover, Kriging was applied to estimate the spatial distribution of negative and positive air ions, as well as the air ion index in the study area. The measurement results showed that the numbers of NAI and PAI differed greatly during the four seasons, the highest and the lowest negative and positive air ion concentrations were found in the summer and winter, respectively. Moreover, temperature was positively affected negative air ions concentration. No matter what temperature is, the ranges of variogram in NAI/PAI were similar during four seasons. It indicated that spatial patterns of NAI/PAI were independent of the seasons and depended on garden elements and configuration, thus the NAP/PAI was a good estimate of the air quality regarding air ions. Kriging maps depicted that the highest negative and positive air ion concentration was next to the waterfall, whereas the lowest air ions areas were next to the exits of the garden. The results reveal that waterscapes are a source of negative and positive air ions, and that plants and green space are a minor source of negative air ions in the study garden. Moreover, temperature and humidity are positively and negatively affected negative air ions concentration, respectively. The proposed monitoring and mapping approach provides a way to effectively assess the patterns of negative and positive air ions in future landscape design projects.

## 1. Introduction

Studies on negative air ions (NAI) began in the 1970s [1,2]. Negative ions in the air were found to act on the parasympathetic nervous system and relax the nerves, whereas positive air ions (PAI) were found to act on and excite the sympathetic nerves [3]. NAI numbers increase under the influence of various factors such as movement of water and water drops, radioactive decay, ultraviolet rays, coronal discharge and lightning [1,2,4]. Waterscapes are a major landscape element in garden landscapes. At some large waterfalls, negative air ions are particularly numerous, reaching several thousands and even tens of thousands per cubic centimeter. On the other hand, in polluted city air, in closed rooms, in moving cars and aircraft, near television sets and computers, the amount of negative air ions falls dramatically to the tens per cubic centimeter [5]. Many cases have demonstrated the beneficial human health effects of negative air ions. Krueger [6] investigated the biochemical mechanisms of the beneficial biological effects of negative air ion inhalation and found it to be useful in decreasing excessive levels of the neurotransmitter serotonin. Wakamura [7] applied NAI to reduce rectal temperature. Nakane *et al*. [8] found that NAI were effective for recovering from the stress caused by computer operation. Conversely, positive air ions can cause excessive stress after brief exposure. After longer exposure, a state of exhaustion can be observed in the form of a lowered norepinephrine level [9].

Researchers have demonstrated that NAI play a role in reducing the transmission of infection in healthcare places [2,10,11] and several authors have reported that NAI inhibit the growth of microorganisms [12]. A space full of excessive NAI is reputed to be associated with quicker recovery from exhausting exercise, sounder sleep, fewer bodily aches and pains, and fewer respiratory complaints [13]. Moreover, some studies have also found that NAI can remove particles from indoor environments [14,15].The types of negative ion generation can be broadly classified as water-generated negative ions and electrically-generated negative ions. Electrically-generated negative ions are produced by the electrical discharge from thunder, radiant energy and ultraviolet light in the natural environment. Air molecules provide the energy which causes electrons to be charged. Discharged electrons combine with oxygen atoms to create negatively-charged air ions [3]. Water-generated negative ions naturally occur around waterfalls and are created by the ionization of water through the Lenard effect [16]. When water breaks up into small droplets, electrons are arranged on the surface of small water droplets in a dipolar fashion [3]. Negatively-charged oxygen molecules combine with several water molecules to form negative ion clusters: O_2_(H_2_O)_n_ [17]. Water-generated negative ions essentially are considered to be a natural source of negative air ions [4]. NAI produced from water have a longer life in air than do electrically-generated negative ions, and they do not adversely affect the body. Water-generated ions exhibit suppression of tumor growth and improvement of cachexia [17]. Many studies have demonstrated that water-generated negative ions are associated with improvements in mood and physical health, and recent research has begun to support the view that negative ions have a net positive effect on health [17–21] and beneficial effects for the respiratory system [22].

Negative ion data could be measured at any place, but they are measured at rather few in actual practice to save time and money. Therefore, data samples are transformed via a series of interpretation steps to acquire complete descriptions of phenomena of interest [23]. A geostatistical scheme is a regular procedure that is an efficient way of mapping according to the stochastic spatial variation. Kriging, a geostatistical method, is a linear interpolation approach that provides a best linear unbiased estimator (BLUE) for quantities that vary spatially [24]. It makes good use of existing knowledge by considering the difference of attribute varies in space through the variogram model [25]. It interpolates algorithms to generate maps of the best local estimate and generally smoothes out the local details of the spatial variation of a particular attribute [26]. Geostatistical methods have been widely applied to simulate the spatial variability of interest in many fields [27–29]. The functions of a residential garden include providing landscape scenery [30], promoting friendship [31], and conferring both physiological [32] and psychological benefits [18]. The role of the garden in human life has been transformed from a source of visual beauty and relaxation, to a source of physiological and psychological health [33]. Thus, the research was to measure and estimate the spatial distributions of negative and positive air ions in a residential garden in central Taiwan using a geostatistical method.

The objective of this research was to understand spatiotemporal changes of air ions and identified sources of air ions in a residential garden. First, negative and positive air ions were measured at thirty monitoring locations in the study garden from July 2009 to June 2010. Then, Kriging was applied to estimate the spatial patterns of the negative and positive air ions and the air ion index in four seasons. Thus, those seasonal interpolate maps enabled an analysis of spatiotemporal changes of negative and positive air ions and the air ion index. We discussed these factors of spatiotemporal air ion changes during the seasons and identified sources of negative and positive air ions in study garden.

## 2. Materials and Methods

### 2.1. Study Area

**Figure 1.**
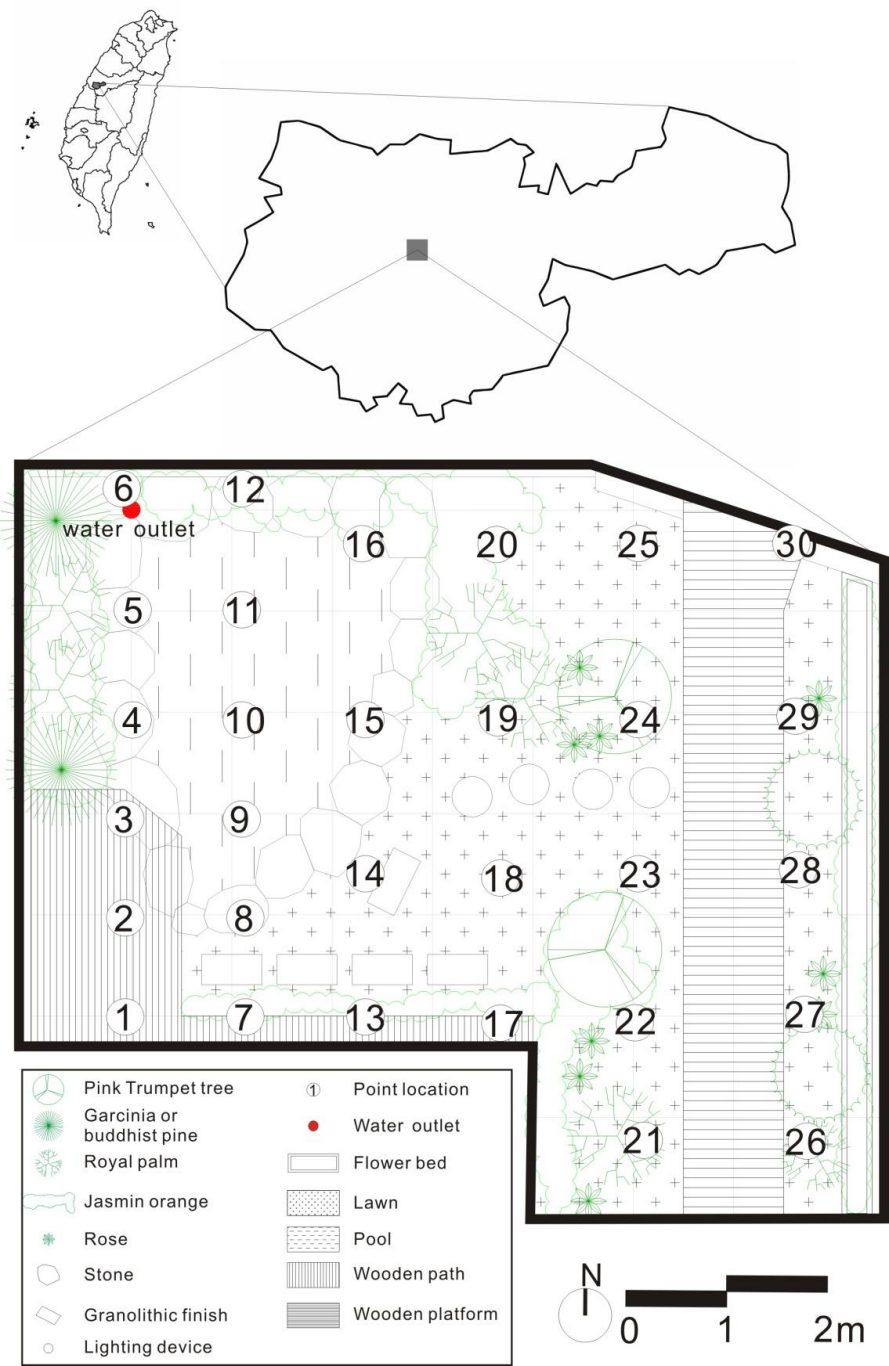
Location and sampling sites of the study garden.

The lifetime of NAI depends on humidity, temperature and other factors [34]. Moreover, the spatial distribution of NAI is affected by their source and the wind speed. Sites selected to research NAI spatial distribution must have similar temperature and humidity, low wind speed and a stable NAI source. In this study, the study area was a residential garden, about 46 square meters in size, located at Taichung city in central Taiwan (Figure 1). It is an enclosed area with a wall about 1.3 meters high located at the eastern and northern sides of the garden. The western side of garden has a building about 27 meters high, and the southern side, a building about 15 meters high. A waterfall in the garden was the NAI source, with the water falling into a pond; both the waterfall and pond are located in the north-west corner. The waterfall is 120 cm high, 80 cm wide, and discharges water at a rate of 0.4 L/s. There is vegetation on the western and northern sides of the pond, a wooden deck on the southern side, grass on the eastern side, and a path for the garden owner in the eastern part.

### 2.2. Sampling and Regression Analysis

Sampling site locations were chosen bearing in mind a homogeneous distribution through the grid and differences in distance to waterfall. 30 sample sites for NAI and PAI monitoring were set in the research garden (Figure 1). Sites 1 to 12 were closer to the waterfall, the NAI source, 1 m apart; sites 13 to 30 were 1.5 meters apart. Two counters, Model ITC-201A (ANDES, Japan), 50 cm high with a tripod stand, were used to simultaneously count the number of negative and positive air ions. Before monitoring, 5 to 15 seconds are needed to stabilize monitor values. After that, each site was monitored for 90 seconds, and every half second the time was auto-recorded. Air ion monitoring was in accordance with the order number of sample sites.

From July 2009 to June 2010, a sunny afternoon of each month was chosen to monitor the air ions at each sampling site from 14 to 16 o’clock. The investigation dates were the 13th of May, 9th of June and 9th of July in summer of 2009, the 13th of August, 10th of September and 12th of October in autumn of 2009, the 12th of November and 10th of December in 2009, and the 10th of January in 2010 in winter, and the 11th of February, 5th of March and 20th of April in 2010 in spring. On each investigated day, at each site, 180 records of numbers of NAI and PAI were collected. The average value of three-month monitoring was the seasonal NAI and PAI value.

In this study, the data was converted to the SPSS statistic version for seasonal descriptive statistics. Monthly air ion concentration was calculated from the mean values of 180 records of NAI, PAI and NAI/PAI. Seasonal average air ion concentration was the mean values of three months of air ion concentration. Moreover, regression analyses were carried out between temperature and humidity with negative and positive air ion concentration, with mean air ion concentration using Microsoft Office Excel.

### 2.3. Variogram and Kriging Estimation

The seasonal NAI, PAI and NAI/PAI values at the 30 sampling sites were used to estimate the spatial distributions in the study garden by the geostatistic method. In geostatistical methods, variograms can be used to quantify the observed relationship between the values of the samples and the proximity of the samples [24]. An experimental variogram for the interval lag distance class *h*, *γ*(*h*), is represented by:

(1) $$\gamma \;(h)=\frac{1}{2n(h)}\sum_{i=1}^{n(h)}{\left[Z(x_{i}+h)-Z(x_{i})\right]}^{2}$$

where *h* is the lag distance that separates pairs of points; *Z*(*x*) is the number of air ions at location *x*, and *Z*(*x+h*), the number of air ions at location (*x+h*) and *n*(*h*) is the number of pairs separated by lag distance *h.*

Kriging is an estimation technique which uses weighted sums of adjacent sample concentrations. The weights depend on the correlation structure exhibited. The weights are determined by minimizing the estimated variance. In this context, Kriging estimates (Best Linear Unbiased Estimators) are the most accurate of all linear estimators. Accordingly, Kriging was used to estimate the value of the random variable at un-sampled location X_0_ based on measured values in a linear form:

(2) $$Z^{*}(x_{0})=\sum_{i=1}^{N}\lambda_{i0}Z(x_{i})$$

where Z^*^(*x*_0_) is the estimated value at location (*x*_0_), *λ_i_*_0_ is the estimated weight of *Z*(*x_i_*), *x_i_* is the location of the sampling point for variable *Z*, and *N* is the number of variables *Z* involved in the estimation.

The NAI and PAI concentration at any of the unsampled sites were determined by geostatistical methods. We assumed that the statistical distribution of sampling data was normal in order to use a geostatistical method. The spatial distribution of concentrations can be characterized by a variogram. The variance is estimated as a function of a variogram model, where the variogram is calculated using the relative locations of the samples. Spatial models were constructed by using three co-ordinates, x, y and z, where x and y are the coordinates of the starting point at the lower left corner of the study garden, and z represents NAI, PAI and NAI/PAI values. The experimental variogram is fitted using a theoretical model, which is spherical, exponential or Gaussian, parameters including the nugget effect (c0), the sill (c) and the range (a) by the software Geostatistic for Environmental Sciences (GS+). c0 is the nugget effect that measures the microscale variation which may be resulted from discontinuousness of the air ions concentration among the sample sites; c is the sill that quantifies the maximum variability of the air ions concentration among the sample sites; a is the range that can be interpreted as the air ions concentration are uncorrelated beyond distance a [18,26,35]. The variogram that best reflected the theoretical model and the highest R square value was chosen. According to these theoretical models, interpolation data was used to obtain air ion concentration maps of the whole area during the four seasons by means of ordinary Kriging.

### 2.4. Constraints for NAI and PAI Investigation

Although, in a previous indoor experiment it was found that the ventilation rate had a minimal influence on ion concentration [12], the spatial distribution of negative air ions in open spaces is easily affected by such factors as precipitation, wind speed, thunder activity and so on, thus making it hard to measure negative air ion distribution in an open space. This study has pioneered research on measuring the spatial distribution of negative air ions in residential gardens. We have focused on the spatial distribution of two dimensions in an open space; as the area of the study garden was only 46 square meters, with walls and buildings surrounding the garden, so the wind and heat flow effects from outside the garden were insignificant. Moreover, the researcher moved slowly in the garden to avoid wind flow in the study area. We have proposed that the wind and precipitation did not affect the spatial distribution of negative air ions. On the other hand, monitoring air ions on sunny days meant that no thunder activity was observed.

## 3. Results and Discussion

### 3.1. Air Ion Statistics and Effects of Temperature and Humidity on Air Ion Concentration

The statistics from the 30 air ion monitoring sites were used to characterize the changes in the number of negative and positive air ions. Table 1 summarizes the statistics for the negative and positive air ions during the four seasons. The lowest mean, standard deviation and coefficient of variation of NAI and PAI occurred during the winter. Moreover, the highest mean, standard deviation and coefficient of variation values of NAI and PAI occurred in the summer. The statistical results showed that the numbers of NAI and PAI differed greatly during these four seasons; the spatial distribution of air ions is more even in the winter. Previous studies have found that air pollution and fog happen more often in winter than in spring or summer. Air ions are easily destroyed by air pollution [28].

**Figure 2.**
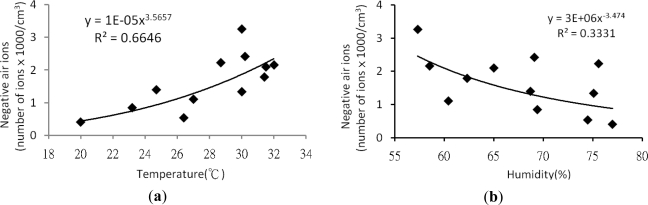

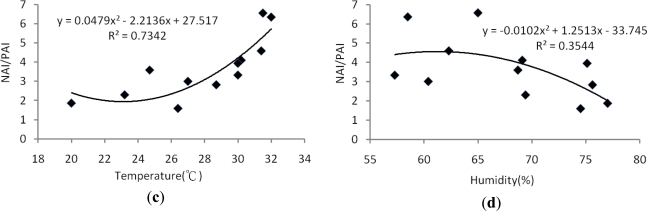
Regression analysis between NAI and NAI/PAI with temperature and humidity Regression analysis between with temperature and humidity. (**a**) NAI with temperature; (**b**) NAI with humidity; (**c**) NAI/PAI with temperature; (**d**) NAI/PAI with humidity.

Many reports have found that temperature and humidity affect the concentration of air ions [13,36]. In this research, regression analyses were carried out between temperature and humidity with NAI, PAI and NAI/PAI using Microsoft Office Excel. Results show that temperature and humidity are positively and negatively related to negative air ion concentration, respectively. Both factors had exponential correlation with negative air ion concentration and R squares were 0.6646 and 0.3331, respectively [Figure 2(a),(b)]. However, the correlation between temperature and humidity with positive air ion concentration were not significant; R square values of exponential regression model were 0.184 and 0.008, respectively. The correlation between temperature and humidity with NAI/PAI show temperature and humidity are positively and negatively related to negative air ion concentration, respectively.

Both factors had binomial correlation with negative air ion concentration and R squares were 0.7342 and 0.3544, respectively [Figure 2(c),(d)]. From those results, it indicated that temperature and humidity were positively and negatively affected NAI and NAI/PAI along the year.

### 3.2. Spatial Pattern Analysis of Air Ions by Variograms

Conclusions of the Kolmogorov-Smirnov normal distribution test of NAI, PAI and NAI/PAI collected at 30 samples site during the four seasons show that all data lacked a normal distribution except PAI in autumn and NAI/PAI in summer. Moreover, the conclusions of a Kolmogorov-Smirnov log-normal distribution test of these collected data showed that only PAI in autumn and winter and NAI/PAI in summer, autumn and winter were log-normal distributions. Therefore, this study assumed that the sampling data were normal distribution for using a Kriging method.

To depict patterns of air ion distribution in the study garden, experimental variograms and their variogram models were first analyzed during the four seasons from autumn in 2009 to summer in 2010 (Table 2). Table 2 shows the parameters and performance of the Kriging interpolations. Both residual sum of squares (RSS) and the R square coefficient provide indicators of how well the model fits the variogram data. The lower the RSS, the better the model fits; and higher the R square coefficient, the better the model fits [37]. The variogram models of the twelve NAI, PAI and NAI/PAI for the four seasons were exponential and Gaussian models. Moreover, the Gaussian models were the most suitable models, except for PAI in the summer and winter. The R square values of all the models were larger than 0.769, except for PAI in the winter at 0.616, which showed that almost all of the models were suitable for the Kriging estimates.

The shape of the variogram was used to understand the spatial structures of NAI, PAI and NAI/PAI. Sill was used to quantify the variability of the air ions concentration among the sample sites. The sill (*i.e.*, spatial variation) of NAI from high to low was: summer, autumn, spring and winter. The sill of PAI from high to low was: autumn, summer, spring and winter. The sill of NAI/PAI from high to low was: spring, autumn, summer and winter. The comparison of sill values during seasons show that high NAI spatial variability in summer, low NAI, PAI and NAI/PAI spatial variability in winter. In addition, Nugget values (*i.e*., variability in local areas) of NAI in spring, summer and autumn were larger than those in winter. Nugget values of PAI during the four seasons were lower than 0.04. Results showed that there was lowest spatial variations of NAI, PAI and NAI/PAI in winter. Moreover, the ranges of variogram in NAI/PAI were similar during four seasons. It indicated that the spatial patterns of NAI/PAI were resembled among four seasons in study area.

### 3.3. Spatial Pattern Estimations of Air Ions by Kriging

**Figure 3.**
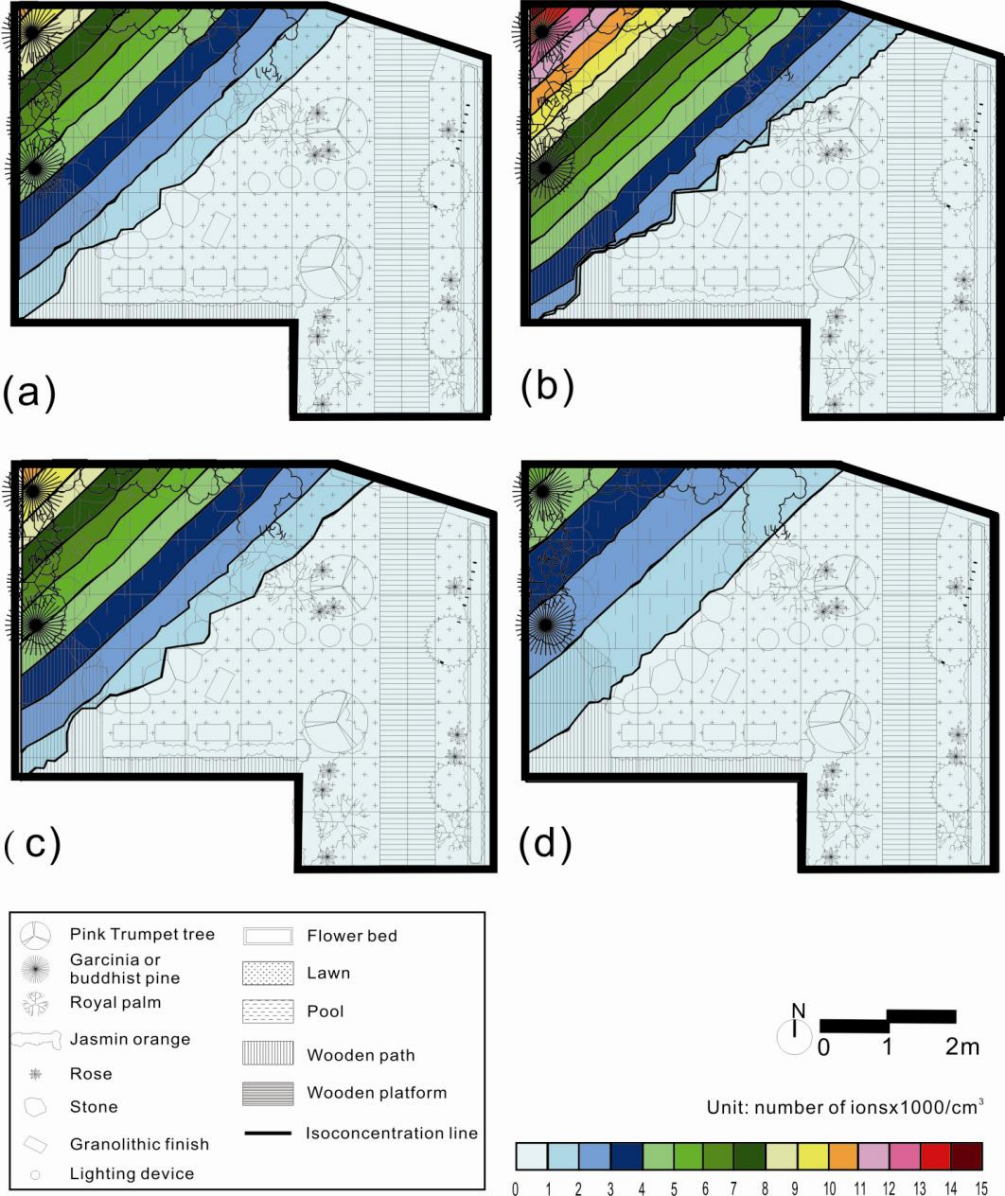
Kriging estimates of negative air ion (NAI) distribution based on 30 samples in the garden during (**a**) spring, (**b**) summer, (**c**) autumn, and (**d**) winter.

In this study, ordinary Kriging estimates were performed based on the above variogram models of 30 samples for the four seasons’ air ion spatial distributions in the research garden. Figure 3 shows the NAI maps produced by the Kriging estimations with the 30 samples from the four seasons. From those maps, it was found that NAI concentration gradually declined according to the distance from the waterfall. The waterfall and pond located in the north-west corner showed the highest NAI concentration during the four seasons. The south-east corner near the building entrance and the wall beside the road had the lowest NAI concentrations during the four seasons. Based on the NAI concentrations during the four seasons, summer had the highest and winter the lowest values in the research garden.

**Figure 4.**
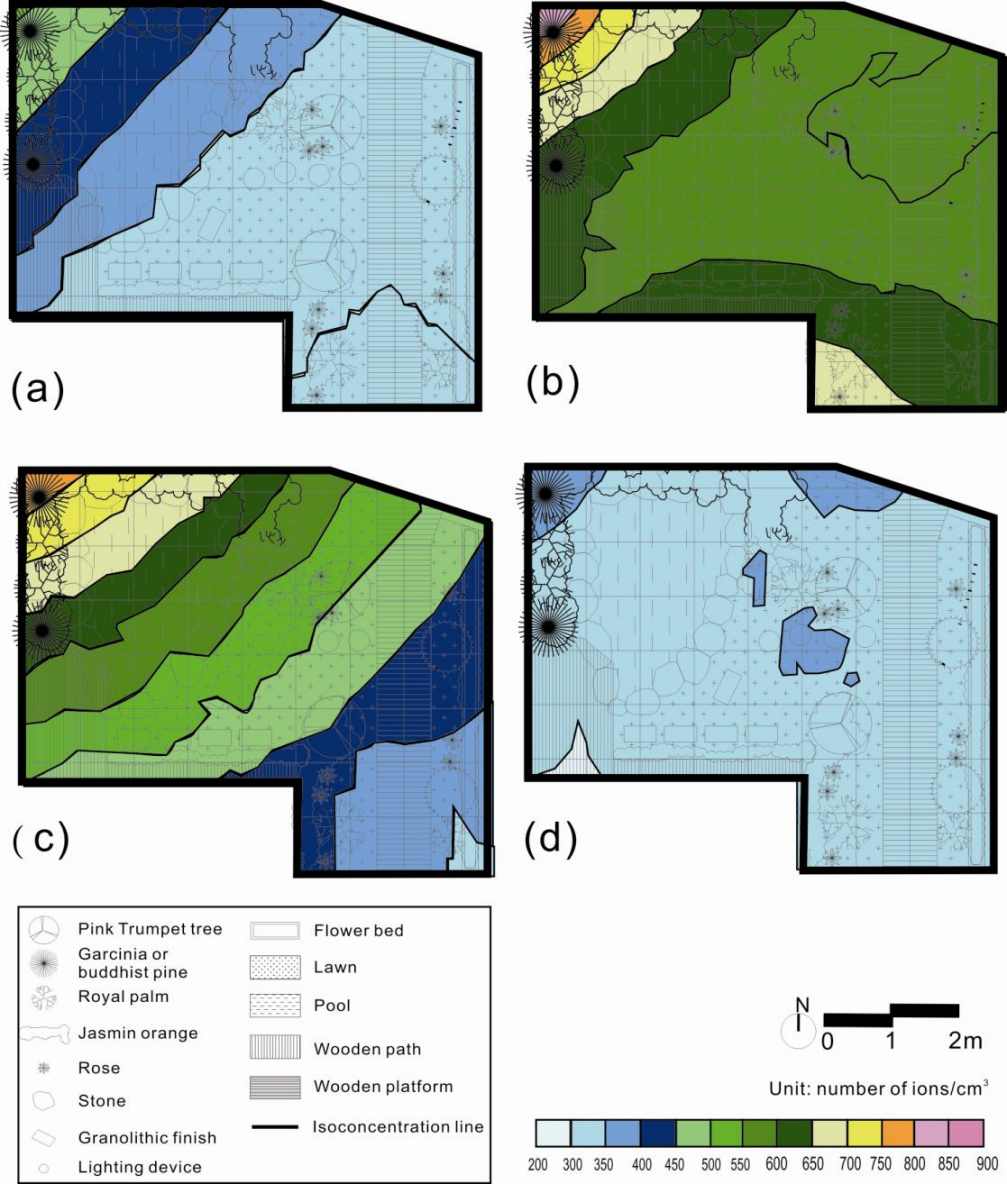
Kriging estimates of positive air ion (PAI) distributions based on 30 samples in the garden during (**a**) spring, (**b**) summer, (**c**) autumn, and (**d**) winter.

Figure 4 showed PAI maps produced by Kriging estimations with 30 samples for the four seasons. From the maps, it was found that PAI concentration gradually declined according to the distance to the waterfall in the north-west corner of the garden. This corner with the waterfall and pond showed the highest PAI concentration in the spring and autumn. The south-east corner near the building entrance had the lowest PAI concentration in both seasons. In summer, the waterfall (north-west corner) and the entrance to the residence (east-south corner) showed high PAI concentrations. In winter, the north-west (waterfall) and north-east (entrance of the garden) corners showed high PAI concentrations. Of the PAI concentrations during the four seasons, the highest PAI concentration distribution in the entire garden was during the summer, and the lowest PAI concentration distribution, during the winter.

**Figure 5.**
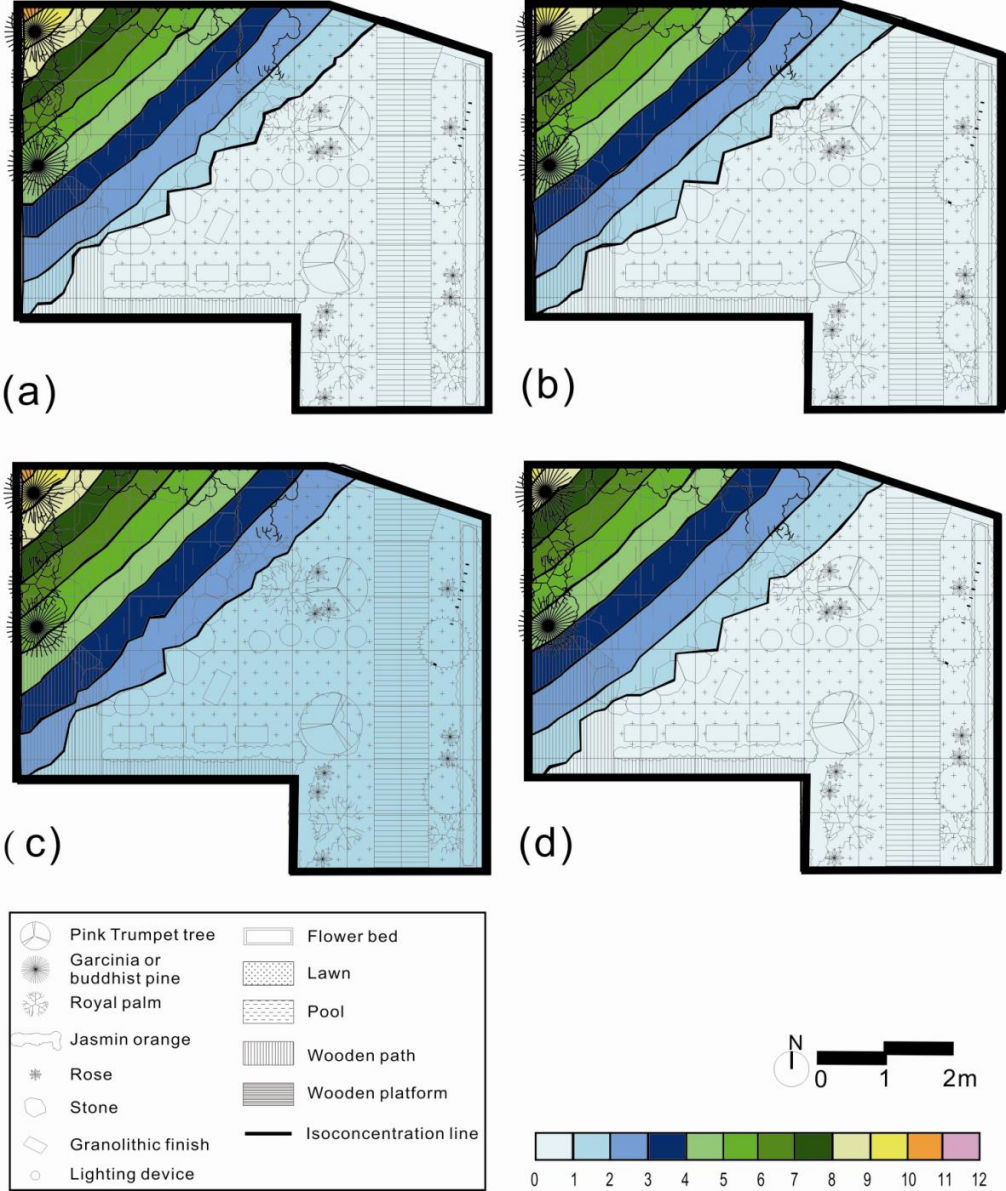
Kriging estimates of negative over positive air ion (NAI/PAI) distributions based on 30 samples in the garden during the (**a**) spring, (**b**) summer, (**c**) autumn, and (**d**) winter.

Figure 5 showed the NAI/PAI maps produced by kriging estimation using the 30 samples from the four seasons. From the four maps, it was found that the value of NAI/PAI gradually declined according to the distance to the waterfall. Similarly, the north-west corner showed the highest NAI/PAI value during all four seasons. The south-east corner had the lowest NAI/PAI value during the four seasons. The spatial distributions of NAI/PAI value among the four seasons were similar. In this finding, the trend of seasonal effect was removed by the NAI/PAI. Thus, NAI/PAI is the indicator of the quality of air ion in the garden design.

The Kriging estimation results illustrated that interpolation techniques, such as Kriging, typically smoothed the view of the distribution of NAI, PAI and NAI/PAI for the four seasons. The Kriging maps showed the highest NAI, PAI and NAI/PAI for the data next to the waterfalls and green spaces in the study garden throughout the four seasons, and the lower NAI, PAI and NAI/PAI areas next to the entrance of the garden and residence. An air ion index, such as the ratio of NAI over PAI, can be an efficient tool for determining the air quality in outdoor environments (Figure 5). In the finding, the trends of seasonal effect will be offset by NAI/PAI. The NAI/PAI is high beside the waterfalls and green spaces but is low at the road entrance. Thus, the landscape elements such as waterfall and green spaces are the driving factors to the quality of air ions.

This study found that the Lenard effect happens near the waterfalls and produces both high negative and positive air ions. Water is an essential reserve value which has an attraction force for landscape planning and design attempts. To provide sustainability in natural and urban areas, suggestions should be considered using waterscape as the key elements in designing and planning [38]. In the study garden, the waterfall is located at the North-West corner; the maximum concentrations amount of waterfall-generated negative air ions were: 38840, 56850, 39890, and 20240 per cubic centimeter during the spring, summer, autumn and winter, respectively. It is demonstrated that the study area is good for human health. On the other hand, green space has positive effects on human health [18,39]. The area around the waterfall and pond in the research garden was planted with garcinia, buddhist pin, royal palm, roses and jasmine oranges. Therefore, the values for both negative air ion and ratio of NAI/PAI are higher near the pond and waterfall than in other areas. Light exerts a positive influence on the generation of negative air ions through plants [40].

Garden designers should allocate more green spaces, plants and waterscapes to increase the NAI concentration. In the design process, the spotted sample sites cannot usually provide sufficient information to highlight the spatial relationships between NAI and landscape elements such as green spaces or waterscapes. The NAI with landscaping elements maps extended the information of sampled points and hotspots areas of NAI. The modeled maps helped to identify that green space, plants and waterscapes were sources of NAI and provide information to lead the public to the good health environment. Therefore, the modeled maps it can be used to identify the locations and suggest the configurations of green spaces or waterscapes, and thereby estimate spatial patterns and concentration of NAI in the future for designers.

## 4. Conclusions

This study has presented an effective approach for integrating air ion monitoring, variograms and Kriging for the efficient evaluation and mapping of the quality of air ions in a residential garden. Systematic air ion monitoring during the four seasons is a useful method for generating spatial and temporal NAI and PAI concentrations in the study area, since NAI and PAI concentration changes induced by the different seasons or a spatial location were easily recognized by comparing the data from the monitoring sites. The measurement results showed that the highest and lowest negative air ion concentrations were found in summer and winter, respectively. Moreover, the highest and the lowest positive air ion concentrations were found in summer and winter, respectively. The statistical results showed that the numbers of NAI and PAI differed greatly during these four seasons; the spatial distribution of air ion is more even in winter. Moreover, temperature was positively affected negative air ions concentration. No matter what temperature is, the ranges of variogram in NAI/PAI were similar during four seasons. It indicated that spatial patterns of NAI/PAI were independent of the seasons and depended on garden elements and configuration, thus the NAP/PAI was a good estimate of the air quality regarding air ions. Kriging maps showed that the highest NAI/PAI areas were next to the waterfall in the study garden while the lowest NAI/PAI areas were next to the entrance of the garden and residence, respectively. The measurement and estimation results showed that the waterscape, plants and green spaces were sources of negative and positive air ions, and that plants were a minor source of negative air ions in the study area. This study has provided a method for effectively assessing and mapping the patterns of negative and positive air ions for future healthful garden design.

## Figures and Tables

**Table 1.** Air ion statistics during seasons.

**Season**	**Air ions**	**Min. ^a^**	**Max. ^a^**	**Mean ^a^**	**SD.**	**CV.**
Spring	NAI	0.035	38.84	1.48	7.06	49.82
	PAI	0.246	1.05	0.45	0.14	0.020
Summer	NAI	0.118	56.85	2.26	10.31	106.32
	PAI	0.421	1.67	0.65	0.23	0.055
Autumn	NAI	0.350	39.89	1.91	7.18	51.48
	PAI	0.181	1.06	0.49	0.21	0.043
Winter	NAI	0.067	20.24	0.88	3.66	13.39
	PAI	0.223	0.65	0.44	0.09	0.008

NAI: negative air ions; PAI: positive air ions; Min: minimum; Max: maximum; SD.: standard deviation; CV.: coefficient of variation;

a: Unit is number of ions × 1,000/cm^3^.

**Table 2.** Variogram models of air ions during seasons.

**Season**	**Air ions**	**Model**	**Nugget**	**Sill**	**Range**	**R^2^**	**RSS**
Spring	NAI	Gaussian model	23.8	108.6	11.102	0.878	286
	PAI	Gaussian model	0.01	0.064	16.403	0.898	2.859E-05
	NAI/PAI	Gaussian model	20.2	101.4	11.432	0.886	222
Summer	NAI	Gaussian model	51.7	314.3	14.29	0.898	1054
	PAI	Exponential model	0.036	0.157	62.97	0.769	1.293E-04
	NAI/PAI	Gaussian model	17.3	84.6	11.258	0.897	143
Autumn	NAI	Gaussian model	24.8	110.6	11.016	0.878	302
	PAI	Gaussian model	0.022	0.245	21.114	0.973	5.166E-05
	NAI/PAI	Gaussian model	22.2	95.4	11.276	0.865	230
Winter	NAI	Gaussian model	6.36	32.71	12.21	0.889	18.7
	PAI	Exponential model	0.006	0.02	62.97	0.616	3.575E-06
	NAI/PAI	Gaussian model	14.3	69.6	11.12	0.895	101

NAI: negative air ions; PAI: positive air ions; NAI/PAI: negative over positive air ions.
